# Effect of perindopril on pulse-wave velocity and endothelin-1 in black hypertensive patients

**DOI:** 10.5830/CVJA-2012-043

**Published:** 2012-08

**Authors:** Elzbieta Osuch, Willem J Du Plooy, Sandra H Du Plooy, Linde H Böhmer

**Affiliations:** Department of Pharmacology and Therapeutics, School of Medicine, MEDUNSA Campus, University of Limpopo, South Africa; Department of Pharmacology and Therapeutics, School of Medicine, MEDUNSA Campus, University of Limpopo, South Africa; Department of Physiology, School of Medicine, MEDUNSA Campus, University of Limpopo, South Africa; Department of Physiology, School of Medicine, MEDUNSA Campus, University of Limpopo, South Africa

**Keywords:** arterial elasticity, pulse-wave velocity, perindopril, endothelin-1, hypertension

## Abstract

**Introduction:**

We investigated the effect of perindopril on pulse-wave velocity (as indicator of arterial elasticity) and endothelin-1 (ET-1) levels in black hypertensive patients.

**Methods:**

Forty-four newly diagnosed hypertensive patients who received 4 mg perindopril daily were monitored for nine months. Pulse-wave velocity (PWV) was measured non-invasively along the carotid–femoral arterial segment (high elastic content) and the brachial–ulnar segment (low elastic content).

**Results:**

There was a significant increase in arterial elasticity, as indicated by a slower PWV in the carotid–femoral segment of the treatment group, from 11.6 to 7.5 m/s after nine months. The PWV of the treatment group (7.5 m/s) after nine months was lower than that of the healthy volunteer group (8.2 m/s) but it was not statistically significant. No correlation between ET-1 and PWV could be found.

**Conclusion:**

In addition to its blood pressure-lowering effect, our study confirmed the improvement in arterial elasticity in patients on perindopril therapy, without involvement of ET-1.

## Abstract

Pulse-wave velocity (PWV) has become the standard for measuring arterial elasticity or stiffness.^1^,^2^ A higher PWV indicates decreased elasticity. The elasticity of the larger arteries ensures a dampening of the pulse wave and it is stored as recoil energy to ensure continuous blood flow, with better perfusion.^3^ PWV was found to be higher in patients with sustained essential hypertension compared to normotensives subjects.^4^

Decreased arterial elasticity and endothelial dysfunction are associated with end-organ damage and, together with pulse pressure, are independent predictors of cardiovascular risk in hypertensive patients.^5^-^7^ Therefore, besides lowering blood pressure, structural and functional vascular properties have become important.^1^,^8^

Only a few studies, however, have reported the effect of different drug therapies on abnormal arterial elasticity. The methods differed widely and the patient numbers varied between 10 and 20.^9^,^10^ In the Complior® study, 4 mg perindopril daily over six months showed an improvement in arterial elasticity.^11^

In patients with congestive heart failure it has been shown that captopril decreased endothelin production.^12^ It has also been shown that increased endothelin-1 (ET-1) was associated with decreased arterial elasticity in hypertensive patients.^13^

In this study we investigated the effect of a nine-month treatment of the angiotensin converting enzyme inhibitor (ACEI) perindopril on arterial elasticity, brachial pulse pressure and the role of ET-1 in black hypertensive patients.

## Methods

Newly diagnosed hypertensive patients with a diastolic pressure of > 85 mmHg and/or a systolic pressure of > 135 mmHg were enrolled in the study. Only treatment-naïve patients were admitted into the trial, after informed consent was obtained.

Patients with secondary hypertension or any concomitant disease were excluded from the study. Those who needed any treatment other than 4 mg of perindopril to control their hypertension were excluded. Patients who were on any chronic or acute medication were also excluded.

Forty-four patients received 4 mg of perindopril daily for a period of nine months. Fifty-one healthy volunteers served as a reference group.

PWV was used as a surrogate indicator of arterial elasticity and was measured non-invasively using a Powerlab 4 SP system (AD Instruments Pty, Ltd, Australia) and connected to a desktop computer. PWV was measured along two segments of the arterial tree, the carotid–femoral segment (representing an arterial segment with a high elastic content) and the brachial–ulnar segment (representing an arterial segment with little elastic content).

The carotid–femoral PWV was calculated from the time delay (∆*t*) between the recorded proximal (carotid) and distal (femoral) beginning of the upstroke of the wave, and the distance (∆*d*) separating the two respective transducers, according to the equation:

speed (*v*) = $\frac{∆d}{∆t}$

Peripheral pulses were detected by miniature infrared plethysmo-Doppler sensors. The same operator placed the sensors, to limit bias. Each recording lasted for 15 seconds and the average of five consecutive pulses was used. Baseline values for volunteers and patients were recorded on three occasions prior to commencement of the study.

Blood pressure was measured using calibrated non-invasive blood pressure (NIBP) equipment (Welch Allyn, Model 5200-103A). Other parameters and measurements included pulse pressure, body mass index (BMI) and lead II of an ECG.

Endothelin-1 was measured using an ^125^I immuno-assay radioactive ligand system (Amersham Biosciences International, South Africa, Cat no RPA 555). Briefly, the assay is based on the competition between unlabelled ET-1 and a fixed quantity of ^125^I-labelled ET-3 (synthetic) for a limited number of binding sites on an ET-1-specific antibody. With fixed amounts of antibody and radioactive ligand, the amount of radioactive ligand bound by the antibody is inversely proportional to the concentration of the added non-radioactive ligand.

The antibody-bound ET-1 is then reacted with a second antibody that is bound to magnetisable polymer particles. Separation of the antibody-bound fraction is effected by centrifugation of the suspension and decantation of the supernatant. Measurement of the radioactivity in the pellet enables the amount of labelled ET-3 in the bound fraction to be calculated. The concentration of the unlabelled ET-1 in the sample was determined from a standard curve. All samples were done in triplicate.

Venous blood (5 ml) was collected into heparinised tubes and centrifuged immediately at 2 000 × *g* for 10 minutes at 4°C to remove the cells, after which the plasma was stored at –70°C for later analysis. Samples were then prepared and analysed according to the manufacturer’s instructions.

Blood was also collected for routine blood analysis, including renin, aldosterone, cholesterol, glucose and electrolyte levels, and liver function. In the hypertensive patients all measurements were done and blood samples collected at baseline (before therapy), and after one, three, six and nine months of therapy.

## Statistical analysis

Analysis of variance (applying the Bonferroni principle) was used to determine intra-group variations at the different intervals. Comparisons between the control (healthy volunteers) and the experimental group (treatment-naïve patients) were done using the Mann-Whitney rank sum test. A *p*-value of < 0.05 was considered significant. Pearson’s coefficient was used to determine correlations between PWV, ET-1 levels and the other parameters. Descriptive statistics were given as median (1st quartile–3rd quartile).

The study was approved by the Medunsa Research Ethics Committee of the University of Limpopo, IRB 00005122.

## Results

Of the 44 newly diagnosed hypertensive patients, 14 were male and 30 female, aged 50.4 ± 7.6 years, whereas in the control group, 19 were male and 32 female, aged 43.2 ± 8.3 years. The BMI at the beginning of the study period was 28 ± 6.9 kg/m^2^ for the control group and 31.5 ± 6.9 kg/m^2^ for the patient group.

Five patients were regarded as lost to follow up, of whom three did not respond to perindopril alone and were give 2.5 mg indapamide, and three patients developed a dry cough. Thirty-nine patients completed the nine-month period on 4 mg perindopril.

There was a significant decrease in systolic, diastolic and mean arterial pressure in the treatment group at each visit compared to baseline, but pulse pressure did not change. Values never reached the same level as those of the control group (Table 1).

**Table 1.** Effect Of Perindopril 4 MG Daily On Blood Pressure In Black Hypertensive Patients After A Nine-Month Treatment Compared To Healthy Volunteers

	*Patients (n = 39)*					
*Variable median (IQF 1–3)*	*Control (n = 51)*	*M0*	*M1*	*M3*	*M6*	*M9*
SBP (mmHg)	116**	149	145*	137*	134*	136*
	(109–127)	(140–154)	(135–150)	(130–150)	(130–146)	(127–149)
DBP (mmHg)	73**	90	90	85	83*	81*
	(65–80)	(85–96)	(85–100)	(80–93)	(77–90)	(75–87)
MAP (mmHg)	88	110	105	99.4	96.6*	97.6*
	(79–96)	(106–113)	(92–113)	(88–108.2)	(85.91–105.4)	(83.9–104.6)
PP (mmHg)	47	55	55	50	50	56
	(39–53)	(50–63)	(45–60)	(43–55)	(47–60)	(49–63)

Values are median IQF 25–75% (1st–3rd). SBP: systolic blood pressure, DBP: diastolic blood pressure, MAP: mean arterial pressure, PP: pulse pressure. *Compared to baseline M0, (M1, M3, M6, M9) after one, three, six and nine months of therapy (*p* < 0.05 Mann-Whitney rank sum test). **Comparison between healthy volunteers (control) and baseline (M0) of patients before treatment. (*p* < 0.05 Mann-Whitney rank sum test).

There was a significant continuous reduction in PWV that could indicate an increase in arterial elasticity in the carotid–femoral segment of the treatment group, from a median of 11.6 to 7.5 m/s over the nine-month period. The PWV of the treatment group (median 7.5 m/s) after nine months was lower than that of the healthy volunteer group (median 8.2 m/s) but was not statistically significant (Table 2).

**Table 2.** Effect Of Perindopril 4 MG Daily On Pwv And Et-1 In Black Hypertensive Patients After A Nine-Month Treatment Compared To Healthy Volunteers

	*Patients (n = 39)*					
*Variable median (IQF 1–3)*	*Control (n = 51)*	*M0*	*M1*	*M3*	*M6*	*M9*
Carotid–femoral PWV (m/s)	8.2**	11.6	9.3	7.96	9.6	7.5*
	(7.0–10.7)	(7.1–14.0)	(6.9–11.5)	(7.4–10.8)	(7.5–10.0)	(6.3–10.5)
Brachial–ulnar PWV (m/s)	6.4 ns	6.4	6.4	6.8	7.2	6.1
	(4.4–9.8)	(5.4–9.5)	(5.3–10.1)	(4.6–8.5)	(4.4–8.8)	(5.0–8.4)
ET-1 (pmol/l)	4.69**	6.15	7.55	7.96	8.15	4.53
	(3.0–5.4)	(3.5–7.89)	(4.66–9.42)	(6.36–8.87)	(5.32–9.63)	(3.68–9.25)

Values are median IQF 25–75% (1st–3rd). *Compared to baseline M0, M1, M3, M6 M9 after one, three, six and nine months of therapy (*p* < 0.05 Mann-Whitney rank sum test). **Comparison between healthy volunteers (control) and baseline (M0) of patients before treatment. (*p* < 0.05 Mann-Whitney rank sum test).

Although the absolute values of the PWV in the brachial–ulnar segment of the treatment group decreased over time, it was not significant. After the nine-month treatment, the average value was lower than that of the healthy volunteer group. The median PWV in the brachial–ulnar segment at all treatment intervals was lower than that of the carotid–femoral segment (Table 2).

All values are given as median (1st–3rd quartile). ET-1 levels in the treatment group first increased from 6.15 (3.5–7.89) pmol/l at baseline to reach a maximum of 8.15 (5.32–9.63) pmol/l after six months but it was not significant. They then decreased to 4.53 (3.68–9.2) pmol/l after nine months.

The ET-1 levels of the treatment group after nine months were lower than those of the healthy volunteer group but it was not significant. The baseline level of ET-1 in the treatment group was significantly higher [6.15 (3.5–7.89) pmol/l] than that of the healthy volunteer group [4.69 (3.0–5.4) pmol/l] (Table 2).

Neither BMI nor other routine blood tests changed during the nine-month study period.

## Discussion

In this study we investigated the effect of a nine-month treatment with the ACE inhibitor perindopril on PWV and the role of ET-1 in black hypertensive patients. PWV was used as a surrogate to indicate arterial elasticity, which includes structure, elastin and collagen.

It has been shown that β-adrenergic blocking agents, diuretics, and some direct vasodilators such as hydralazine and dihydralazine, only lowered blood pressure but had no effect on vascular elasticity, whereas the ACE inhibitors, calcium channel blockers in general and nitroprusside, in addition to their blood pressure-lowering properties, also improved arterial elasticity.^10^,^14^ In a six-month study it was shown that perindopril improved arterial elasticity in hypertensive patients independent of its blood pressure-lowering properties.^11^

In a number of studies, possible mechanisms were investigated. It was suggested that vascular collagen metabolism plays a role. However, no correlation could be found between matrix metalloproteinase (MMP)-1, the tissue inhibitor of MMP-1 (TIMMP-1), PWV and blood pressure in patients who received perindopril for six months.^15^ A more recent study showed that ET-1 contributed to a decreased arterial compliance in hypertension through inhibition of collagen degradation.^13^ Furthermore, it has been shown that captopril therapy in congestive heart failure decreased endothelin production.^12^

However, in our study, no correlation could be found between ET-1 levels and arterial elasticity in any arterial segment or at any of the measured intervals. This was in contrast to a study done in endurance-trained men, where a linear correlation between ET-1 and aortic PWV was found.^16^

The segment in which the arterial elasticity is measured is important. In a previous study it was found that perindopril had a smaller effect on the elasticity in the carotid artery than the femoral artery.^17^ In contrast to our study where the arterial elasticity in the brachial–ulnar segment was not affected by perindopril, another study showed an improvement in brachial elasticity.^18^

## Conclusion

In addition to its blood pressure-lowering effect, our study confirms the improvement in arterial elasticity in the carotid–femoral segment but not the brachial–ulnar segment in patients on perindopril therapy. Furthermore, we have shown that ET-1 was not correlated to arterial elasticity in patients receiving perindopril.

As referenced by Milan *et al.*, the European Society of Hypertension has now included in their guidelines the improvement of arterial elasticity as one of the therapeutic aims in the treatment of hypertension.^19^
